# Proactive Management of the Equine Athlete

**DOI:** 10.3390/ani2040640

**Published:** 2012-12-19

**Authors:** Chris W. Rogers, Charlotte F. Bolwell, Erica K. Gee

**Affiliations:** Massey Equine, IVABS, Massey University, Private Bag 11222, Palmerston North, New Zealand; E-Mails: c.bolwell@massey.ac.nz (C.F.B.); e.k.gee@massey.ac.nz (E.K.G.)

**Keywords:** horse, lameness, DOHAD, rehabilitation, exercise, workload, management, racing, show jumping, dressage

## Abstract

**Simple Summary:**

The athletic career of a horse is relatively short. Career length can be positively influenced by the trainer and the age at which the horse starts competition. There are opportunities for a team approach of health professionals and changes in management to improve functional/competition life. The ability to improve the tolerance of the tissue to exercise load via the introduction of early exercise, which reflects the horse’s evolutionary cursorial lifestyle, could provide a proactive mechanism to attenuate injury risk.

**Abstract:**

Across many equestrian disciplines the median competition career of a horse is relatively short. One of the major reasons for short career length is musculoskeletal injury and a consistent variable is the trainer effect. There are significant opportunities within equestrian sport for a holistic approach to horse health to attenuate musculoskeletal injury. Proactive integration of care by health professionals could provide a mechanism to attenuate injury risk and the trainer effect. However, the limited data available on current exercise regimens for sport horses restricts interpretation of how management and exercise volume could be modified to reduce injury risk. Early exercise in the juvenile horse (*i.e*., pre weaning) has a positive effect on stimulating the musculoskeletal system and primes the horse for an athletic career. The early introduction to sport competition has also been identified to have a positive effect on career length. These data indicate that management systems reflecting the cursorial evolution of the horse may aid in attenuating loss from sport due to musculoskeletal injury.

## 1. Introduction

Across all equestrian disciplines, one of the most consistent and major reasons for the loss of animals is in relation to lameness and musculoskeletal injury [1,2,3]. There is a strong relationship between the horse’s athletic ability and its resulting economic worth [4], with a lack of athletic use ultimately impacting on the horse’s functional life [5]. Functional life or longevity, which has most often been studied, does not always reflect true longevity but does accurately reflect commercial life [5,6,7]. Irrespective of discipline, functional life within equestrian sport appears to be limited to a median of approximately 4 years [6,7]. The length of functional life is moderated by a number of variables, one of the most prominent being the positive association of an earlier start of competitive career with the longer resultant competition career length [6,8,9,10]. This positive association may be due to earlier stimulation of the musculoskeletal system in preparation for the future demands placed on it for an athletic career [11,12,13].

Within (elite) equestrian sport much attention is paid to maintaining orthopaedic health and maximizing athletic career. However, in somewhat stark contrast to the integration observed between healthcare practitioners within human sports, there appears to be poor integration or even a lack of a holistic approach to managing the equine athlete [14]. Surveys within the Netherlands and New Zealand have identified that many riders/trainers have a fractionated approach to managing horse health and there is limited communication between primary veterinarians, team veterinarians and allied health practitioners [15,16,17].

The limited functional career and the significant effort placed on maximizing orthopaedic health and competitiveness in (elite) equestrian sport has led to some authors and breed organizations identifying that durability, or career longevity, is a parameter that should be included within the breeding objective and actively selected for [6,7]. The heritability of longevity in horses has been previously estimated [6,7] and indicates that while there is a genetic component to the trait, most of the phenotypic variation is due to the environment or non-genetic factors. As a result, there is significant scope to modify these factors to alter and improve functional longevity in the athletic horse. 

The non-genetic component of functional longevity can be modified reactively or proactively. Much of the attention of clinicians and allied health practitioners involves the reactive management at the “coal face” once the horse is in work as an athlete. With our current understanding of the Developmental Origins of Health and Disease (DOHAD) and the plasticity of tissue to stimuli early in life, the opportunity exists to alter susceptibility to injury at the tissue level. This can be done early in the growth and development phase of the horse, prior to, or at the early stages of, the athletic career [10,11,13,18,19]. 

The objective of this review is to describe the production of the equine athlete in relation to the potential impact on musculoskeletal health, and the opportunity for proactive management to attenuate injury as a result of athletic use. 

## 2. Longevity or Functional Life

In comparison to other major livestock species the value and use of the horse has revolved around its role as a draft and transport animal rather than production for food or fibre. With the advent of mechanization this role has shifted from work to activities ranging from a purely leisure/recreation activity to highly demanding professional sport, with most horses in the western world being riding (sport) horses, competing at various levels [20,21]. This change in use has been reflected in an increase in both the economic and marginal utility of the horse. This transition has also been associated with the reintroduction of sectors of society to the horse and a greater need for education relating to the management and production of horses [20]. Generally, new entrants to equestrian sport tend to have the educational and financial means to actively upgrade their skills and this provides an opportunity for an integrative approach to horse management, injury prevention and rehabilitation with a variety of equine professionals. 

### 2.1. Lifespan Domestic *vs.* Feral/Wild Populations

Estimates of functional life can be obtained directly from insurance records or indirectly from performance and registration records [22]. However, estimates of “true” longevity, or lifespan, are harder to come by due to lack of census demographic data for horses in most western countries. Data from Sweden indicates that the median lifespan of Warmblood (riding) horses were 14.7 ± 0.5 years for males (geldings and stallions) and 22.2 ± 1.2 years for mares [5]. These data are less than the common estimate of lifespan of the horse as 30–40 years [23], but this figure is biased upwards with the inclusion of ponies, which are overrepresented in the older categories of many cross-sectional surveys [24]. Data from cross-sectional surveys are generally consistent in the proportion of “aged” or geriatric horses within the domestic population between 29–33% [23,24,25]. It is believed that the proportion of aged or geriatric horses is increasing within western countries [26] reflecting the greater emphasis on marginal or emotional value rather than productive measures of value or utility.

In the domestic population just under 60% of survey respondents knew the exact age of the horse [24]. In a feral herd estimation of age becomes increasingly complex with the reliance on dental records to differentiate age of animals unless accurate prospective data is captured or complete census regularly undertaken [27]. This limitation often leads to the broad classification of feral populations into juvenile, sub adult and adult (usually >4 or 5 years). Mortality is often greatest in juveniles (13%) but decreases to 3–5% in sub adult and adult age classes [27]. The mortality figures for juveniles in feral populations were lower than those reported for extensively managed domestic horses (22%) [28] but within the ranges reported for intensively managed Thoroughbred foals (4.7–11%) [29,30]. However, cross-sectional data from abattoirs in Europe indicate a non-linear association of horse euthanasia with age, and a clustering of horses sent to slaughter in the 2–7 age group indicating most loss is associated with loss of functionality for intended purpose [31]. 

### 2.2. Functional Life

This issue of longevity or functional life was first identified within the production animal species due to the economic impact of high turnover of animals. The economic importance of longevity led to the identification of a genetic basis and the inclusion of longevity traits within the breeding objective and selection of dairy cattle [32]. Recently, a number of publications have reported that within horses, similar to these other production animals, there is a genetic component to longevity or functional life [6,7]. The reported heritability for longevity in horses lies between 0.07–0.17 [6,7], which is within the range of heritability reported for other traits of economic importance such as show jumping and dressage performance [33]. A number of breed organisations have durability within their reported breeding objectives [34] and it would appear likely that longevity as a discrete trait will be actively included within the selection procedures for breeding stock at some stage. However, the relatively moderate heritability value of longevity or functional life demonstrates significant potential for the modification of the non-genetic component of this trait, through manipulation of the early rearing environment and management practices.

### 2.3. Estimates of Longevity

There have been a number of studies that have investigated the functional life of racing and sport horses [8,9,10,35,36,37]. The resulting estimates of functional longevity are similar across equestrian sports, with surprisingly limited variation in the estimates obtained from a number of countries. Within racing, estimates of longevity are 2-3 years for flat racing Thoroughbreds and for harness racing Standardbreds [9,10]. Within dressage, show jumping and eventing the estimates of longevity are only slightly greater at 3–4 years [8,35,36,37,38]. One of the most consistent associations identified across all studies has been the longer functional career associated with an early start to training or competition. This pattern has been described in racehorses, with those that started as 2-year-olds having longer and more successful careers than those that started later in life [9,10]. A similar pattern has also been observed in equestrian sports with a much later early career start, such as show jumping [6,7,8,9,10]. 

Within a riding club environment insurance data provide an opportunity to examine functional life, perhaps approximating that of many non-elite sport horses. Data from Swedish riding club insurance claims indicated a median of approximately 5 years before the first insurance claim, with 20% of the population having a life insurance claim within a five year observation period [39]. 

## 3. Reason for Loss from Sport

Epidemiological studies have provided a clear indication of the major reasons for wastage and injury in racing and riding/sport horses. However, in contrast, there are limited data on the injury rate and types of injuries sustained by horses in a feral environment. This is understandable given the difficulty in collecting such data prospectively, and the dramatic effect changes in environmental conditions can have on seasonal mortality rates [40,41]. In a population of reintroduced Prezwalski horses approximately one third of the re-introduced herd died in the first 3 years of release. Post mortem findings attributed many of the deaths to equine piroplasmosis and S*treptococus equi equi* infection, both of which are manageable animal health issues in a domestic environment [42]. Abattoir surveys from horses systematically culled from the New Zealand Kaimanawa feral horse population have provided data to demonstrate presence of osteoarthritis in the metacarpaophalangeal joint, the severity of which increased with age, similar to that observed in racing and sporting horses [43]. There was also evidence of a high prevalence of chronic laminitis (45%) within this population identifying that laminitic insult is not just a problem associated with the domesticated horse [44,45].

Across the equestrian sports, the major reasons for loss or culling (end of functional life) from the population are alike, and they have similar prevalence. Musculoskeletal injury consistently accounts for most loss, followed by disorders of the respiratory and the gastrointestinal system. Within racing, prospective studies have identified that musculoskeletal injuries account for 56–79% of lost training days and approximately 25% of reasons for loss from the industry [46,47,48,49]. This pattern is repeated across the equestrian sports with musculoskeletal injury accounting for approximately one third of all losses and wastage within dressage, show jumping, and eventing [3,5,50].

The consistent proportion of losses due to musculoskeletal injury does not reflect the varied mix of sport-specific musculoskeletal injuries. This variation or specificity of injuries is due in part to the age at which peak performance is achieved and the nature of the loading on the musculoskeletal system. Flat racing provides the greatest contrast in age-specific injuries with shin soreness, or dorsal metacarpal disease, being associated with the initial race preparation of juvenile racehorses [48]. In contrast, injuries to the tendonous structures, principally the superficial digital flexor tendon (SDFT), are clustered among the older staying racehorses [51,52].

Within equestrian sport there appears to be a trend for differentiation of injury predilection sites and types of injures between elite and non-elite level horses [53]. In a survey of dressage horses [46], 33% had experienced some lameness resulting in a median of 3 months off training and 5 months off competition. The highest proportion of lameness was found in the Grand Prix and Intermediate II group, confirming the performance level effect identified in an earlier publication [3,53]. The training practices of dressage horses appear to place them at risk for hind limb suspensory ligament injury, which may be a repetitive strain type of injury. Back problems also appear to have a relatively high prevalence with 40% of the dressage horses reported to have had a back problem in the last 2 years, with most of these cases (63%) treated with complementary therapy, which reflects similar data for use of allied health practitioners over different disciplines within New Zealand [15,17]. 

In contrast to the data available for dressage horses, the peer-reviewed literature for show jumping and event horses is relatively sparse. The prevalent injuries described in show jumping horses are in the distal limb and relate to concussive injury and age related changes in tendonous support structures [2,53]. Similarly, event horses appear to be at greater risk for strain/fatigue injuries such as SDFT tendonitis [2,8] 

Prospective and retrospective epidemiological studies have consistently identified a number of management variables associated with lameness and wastage across disciplines. Rider/trainer as a factor are often significant, which signifies that some holistic measure of the unique composition of the management and training of horses predisposes some to injury while other practices are protective [54,55,56]. Data from Swedish riding schools helps elucidate some of the trainer/rider variables that could be associated with greater risk of injury and loss. Significant differences existed between riding schools with high and low insurance claims for orthopaedic injury in relation to the length of work experience of managers and the structure of the introduction to work for new horses. However, there were no significant differences between groups for the volume of work [57]. Both protective parameters indicate an experience component and an opportunity for proactive management by an equine sports medicine support team (clinician and allied health practitioners) to assist young or junior rider/trainer/managers with the development and implementation of workload programmes. The return on such a holistic approach would be two-fold given the evidence that trainer effect not only influences risk of injury but also the time to recovery [1]. 

Despite the widespread use and integration of a holistic approach to managing human elite athletes the same cannot be routinely said of the equine athlete. Often health practitioners all contribute to the management of the equine athlete but there is little, or no, communication between practitioners. This fractionated approach means that the pathophysiology is often quantified and managed independently of the associated adaptive changes and causative factors [56]. The common association between back pain and lameness is a good example of the benefit from an integrative approach [15,58,59]. Allied health practitioners are often the first point of contact for suspected back pain, which often a resultant response to lameness. Integration of health practitioners in management of horses with such conditions would permit resolution of the causative factors and application of an appropriate rehabilitation programme to resolve associated negative musculoskeletal response. 

## 4. Can Managing the Horse as a Horse be a Proactive Response?

Evolutionary the horse is a cursorial creature and comparison of locomotory activity between domestic/conventionally managed horses and their feral counterparts has identified significant differences [60]. Whereas feral horses will travel 8.1–28.3 km/day, a horse kept within a 6 m × 6 m yard (which is larger than the EU standard of 3.6 × 3.6 m) is estimated to travel only 1.1 km/day [61]. The exercise/distance covered when kept in a yard is exclusive of the workload of the horse when prepared for competition or when regularly ridden. However, this is where it becomes difficult from a proactive management perspective to plan what is optimal workload, or more precisely the workload volume, for a given horse. Furthermore, should daily distance covered / exercise volume be the same as those observed with feral horses?

Despite the high economic and marginal utility value of elite sport horses there are limited data on current management and training practices [62,63]. This is not surprising given the lack of detailed data for racehorses even though they have relatively less varied and complex training programmes that are easier to measure/quantify than sport horses [64,65]. The lack of workload data within the equine racing industry may stem from the competitive nature of the industry, in which performance is measured by competing ahead of other individuals. In racing terms a small improvement in 100ths of a second racing time can mean the difference between winning and not being placed. Additionally, a variety of measures are used to quantify workload that appear dependent on the researcher and the organ system of interest making comparisons difficult, though most utilize some measure of load (HR_max_ or speed) and exposure (time or distance travelled) [66,67,68].

Within the racing environment it is relatively easy to quantify workload with the use of Global Positioning Satellite (GPS) and heart rate (HR) monitors, which are increasingly being used by commercial trainers [69]. In contrast, the uptake of this technology within other equestrian sports does not appear to be as rapid and estimates of workload rely on duration and semi objective scoring of workload effort or difficulty [62,63,70]. This may be a result of the greater schooling rather than conditioning aspect to other equestrian sports and that much of the exercise is at sub maximal levels.

Across a variety of equestrian sports the duration of ridden activity/daily exercise reported was consistently less than 45 minutes per day [62,70,71]. Currently, we lack the data to determine if this is the optimal exercise level to proactively reduce orthopaedic injury. At present, some data reported on exercise parameters may indicate the need for changes in training management [3]. However, such data may be in response to pre-existing conditions rather than risk factors for injury. These results highlight the need for prospective data capture to further refine these risk factors [3]. Studies examining the ability of the horse to self-select the appropriate exercise load appear to have been limited by some of the behavioural aspects of the horses, in particular the drive for social contact and grazing, which appear to override self-selected exercise choices [72,73].

### 4.1. Proactive Management Opportunities During Growth and Development

Much of the attention with the athletic horse is focused on management at the competition level. However, there is the opportunity to attenuate the injury risk with appropriate stimulation of the tissue during growth and development. Within the human literature there is a considerable body of evidence to demonstrate plasticity of tissue to stimuli, *in utero* and in the early neonatal phase [18]. The Barker or Developmental Origins of Health and Disease (DOHAD) hypothesis proposes that manipulation of the perinatal environment, including but not restricted to the maternal plane of nutrition, can alter the genotypic expression of the developing foetus resulting in a persistent change in phenotype and or susceptibility to chronic disease. Within the horse there are some data indicating fetal perinatal plasticity to fetal or maternal nutritional challenges. In line with results from other mammals maternal nutritional challenges to the mare evoked enhanced pancreatic beta cell sensitivity to glucose and were proposed to predispose the foals to metabolic problems later in life [74]. 

Within the domestic environment the maternal nutritional challenges tend to be over rather than under-feeding. Thoroughbred broodmares are often fed high starch and soluble carbohydrate rations during pregnancy to maximise fetal growth [74]. However, literature from other mammalian species indicates a negative association of high energy maternal diets on placental and foetal growth [75] and a predisposition for reduced insulin sensitivity in offspring [76]. At 160 days of age foals from mares receiving a high starch diet exhibited a trend for lower insulin sensitivity indicating metabolic programming [77]. In the horse insulin resistance has been associated with equine metabolic syndrome, the development of laminitis and with the presence of osteochondrosis in the growing horse [78,79]. 

A discrete example of the effect of maternal nutrition on subsequent musculoskeletal development is the cases of congenital hypothyroidism and dysmaturity (CHD) first reported in western Canada [80]. High nitrate greenfeed provided to mares during gestation was a significant risk factor (Odds Ratio (OR) 13.1) for CHD foals as was the absence of supplemental salt or mineral during the winter (OR 5.6) [81]. These data demonstrate how subtle modifications in maternal nutrition can provide significant alternations in the foals phenotype. 

Across mammalian species there is evidence that early (*i.e.*, pre-pubescent) exercise can modify organ systems and metabolic pathways to generate effects as diverse as improving muscle mass and bone mineral density, increasing the resistance to obesity and delaying the onset of diabetes [82,83,84]. Within the equine literature perhaps the best documented response to early exercise has been the effect on cartilage within the distal limb. The neonatal foal is born with a “blank joint” and cartilage heterogeneity occurs in response to exercise stimuli [85]. The foal therefore requires sufficient high intensity exercise early in life to stimulate biochemical heterogeneity, and this appears to occur naturally. Previous observations indicate that workload in foals in the first month of life was approximately twice that of older foals and high speed activity decreases with increasing age [86,87,88]. Cartilage may also be receptive to additional stimuli over and above that typically available to foals with free exercise at pasture. Thoroughbred foals provided with 30% greater workload than that available to conventionally reared controls demonstrated greater chondrocyte viability and an advanced maturation of the extracellular matrix [19,89,90]. In the same Thoroughbred foals the application of early exercise was associated with increased strength in the third metacarpal diaphysis, but also with significantly larger size of the third metacarpal distal epiphysis [12,13]. The authors proposed that this response within the distal epiphysis could provide the metacarpophalangeal joint with a greater tolerance to the high strain rate loads applied during race training.

Appropriate exercise early in life may also have beneficial effects that may attenuate injury, beyond stimulating tissue maturation. In a cohort of warmblood foals, early introduction to free jumping at 6 months of age resulted in 4-year-old horses that jumped more efficiently and with less intra-individual variation [91]. The implication of this for injury relates to the reduced variation in load and increased efficiency, as forelimb loading patterns vary greatly between horses and appear very dependent on technique [92]. The addition of a rider also complicates the issue, as it is proposed that the load on the distal limb with the addition of a rider increases and delays the timing of the peak load [93]. The interaction of locomotory efficiency and injury was highlighted in a small study of 2-year-old racehorses where early preconditioning was associated with a delay in quantifiable changes in gait associated with high speed loading of the metacarpophalangeal joint with race training [94]. These findings indicate that early exercise does not only have just a discrete positive effect on development but perhaps may have a “whole animal” effect ranging from tissue development to neuromuscular control and coordination, all of which would have a role in attenuating injury risk.

### 4.2. Welfare and Public Perception of Equestrian Sport

Preventative animal health programs aim to minimise losses due to preventable disease. These programs have been very successful and therefore musculoskeletal injury becomes the major reason for loss, or wastage, within most equestrian industries [2,47,48,55]. Thus improvement in the treatment and prevention of musculoskeletal injury have become a key area of attention for the veterinary and scientific community but also for lobby groups interested in animal welfare [95].

Modification of the equine production system to optimise musculoskeletal health has a synergistic benefit for the horse and the equine industry. Improvement in musculoskeletal health improves the stability and efficiency of the production systems by attenuating both biological (the horse) and financial wastage. The goal of optimising horse welfare is a common objective or aim stated by many of the governing bodies for racing and equestrian sport [96]. However, often infrequent but dramatic events of musculoskeletal injury, such as the catastrophic breakdown of racehorses, can evoke strong negative associations of racing and animal welfare [95]. At a lower, but perhaps of more sustained, level of wider public awareness are the negative public perceptions of training techniques such as “Rolkur” [97]. Opponents of this technique propose that it compromises the psychological and musculoskeletal health of the horse [97]. 

A common theme to many of the negative public perceptions of racing and equestrian sport are the demand for partnerships in alignment with the horses’ physical, mental and evolutionary constraints rather than exploitation. Altering the early rearing and production system to optimise musculoskeletal health addresses the need of the horse, the industry and the concerns of the animal welfare lobby; providing alignment of the industry with a harmonious partnership with the horse rather than a sometimes perceived role of exploitation. 

### 4.3. How to Influence Management and Health Care

Participants within equestrian sport generally have the financial and educational ability to actively upgrade their equine skills and knowledge. Education provides a mechanism to disseminate recent research findings to the end-users of the information for rapid uptake. The challenges lie in the appropriate medium to rapidly and effectively disseminate research led education. Across a number of surveys veterinarians were consistently identified as a primary source of information [98]. The difficulty lies in the lag between dissemination of research information, uptake by the veterinarian and dissemination to their client. Traditional education pathways tend to be relatively rigid and have an inherent latency. With the large participation of society with social media this could provide a rapid and effective mechanism to disseminate research led education [99]. 

Legislative processes to modify equine management provide a robust framework for acceptable practise, with many countries using these to provide minimum standards of care and management for animal welfare. However, they are relatively conservative, inflexible, and resistant to change due to the latency involved in the legislative process. This difficulty means that legislation is best suited to provide framework for minimal levels of care, rather than a process to permit optimal management by dynamically integrating recent research.

In line with changes in human medicine the increasing economic and emotional value of the horse has seen a greater emphasis placed on proactive optimising of welfare and performance rather than reactive treatment of clinical signs of disease [16]. The recently introduced American College of Veterinary Sports Medicine and Rehabilitation diplomate qualification recognises the central role of these veterinarians in optimising equine health and performance [100]. These changes place the veterinarian within a pivotal role to provide a framework for the integration of the allied health practitioners. Leading equine practices and University clinics no longer fractionate care but integrate the roles of the farrier, physiotherapist and clinician to address not just the symptoms but to holistically and proactively address the probable causative factors in the management and training of that horse. 

## 5. Conclusions

At present the competition career, or functional life, of an equine athlete is comparatively short and in comparison to elite human sport there appears to be limited integration of clinicians and allied health practitioners in management or care of the equine athlete. The consistent rider trainer effect within epidemiological studies indicates the potential to utilise a holistic equine sports medicine team to proactively attenuate injury risk and reduce time out of training and competition due to injury. Limited data on current exercise regimens for sport horses restricts interpretation of how management and exercise volume could be modified to reduce injury risk. Alteration of the early growth and exercise environment of the developing horse could stimulate tissue development. The positive effect of early exercise on tissue development does not appear to be restricted to the period of rapid growth but may also be effective with an earlier introduction of the horse to the training and stimuli required for racing and equestrian sport, once the period of most rapid growth and development had been completed. 
